# LOCATE-US: Indoor Positioning for Mobile Devices Using Encoded Ultrasonic Signals, Inertial Sensors and Graph-Matching

**DOI:** 10.3390/s21061950

**Published:** 2021-03-10

**Authors:** David Gualda, María Carmen Pérez-Rubio, Jesús Ureña, Sergio Pérez-Bachiller, José Manuel Villadangos, Álvaro Hernández, Juan Jesús García, Ana Jiménez

**Affiliations:** 1 Department of Electronics, University of Alcalá, Alcalá de Henares, 28801 Madrid, Spain; sergio.perezb@uah.es (S.P.-B.); jm.villadangos@uah.es (J.M.V.); alvaro.hernandez@uah.es (Á.H.); jjesus.garcia@uah.es (J.J.G.); ana.jimenez@uah.es (A.J.); 2 Signal Theory and Communications Department, King Juan Carlos University, Móstoles, 28933 Madrid, Spain

**Keywords:** indoor LPS, smartphone positioning, ultrasonic signals, inertial measurements, sensor fusion, Android application

## Abstract

Indoor positioning remains a challenge and, despite much research and development carried out in the last decade, there is still no standard as with the Global Navigation Satellite Systems (GNSS) outdoors. This paper presents an indoor positioning system called LOCATE-US with adjustable granularity for use with commercial mobile devices, such as smartphones or tablets. LOCATE-US is privacy-oriented and allows every device to compute its own position by fusing ultrasonic, inertial sensor measurements and map information. Ultrasonic Local Positioning Systems (U-LPS) based on encoded signals are placed in critical zones that require an accuracy below a few decimeters to correct the accumulated drift errors of the inertial measurements. These systems are well suited to work at room level as walls confine acoustic waves inside. To avoid audible artifacts, the U-LPS emission is set at 41.67 kHz, and an ultrasonic acquisition module with reduced dimensions is attached to the mobile device through the USB port to capture signals. Processing in the mobile device involves an improved Time Differences of Arrival (TDOA) estimation that is fused with the measurements from an external inertial sensor to obtain real-time location and trajectory display at a 10 Hz rate. Graph-matching has also been included, considering available prior knowledge about the navigation scenario. This kind of device is an adequate platform for Location-Based Services (LBS), enabling applications such as augmented reality, guiding applications, or people monitoring and assistance. The system architecture can easily incorporate new sensors in the future, such as UWB, RFiD or others.

## 1. Introduction

Development of services and applications based on contextual information is a current necessity. The widespread use of mobile devices, such as smartphones or tablets, that can connect to the internet in any place and at any time, has led to an enormous variety of potential commercial applications such as advertising, gaming, social networks, augmented reality, emergency assistance and indoor routing [1]. Thus, there are numerous markets and sectors that are waiting for a technical solution to the problem of estimating accurately and in real time positions in places where global navigation satellite systems (GNSS) signals are not available, such as indoor environments (museums, hospitals, malls, airports and many others). In some of these indoor environments there are maps available. Google, which is one of the most prominent worldwide maps providers, offers indoor mapping for many public buildings through Google Indoor Maps. Nevertheless, the estimated position achieves an accuracy of tens of meters with a high uncertainty, since it is based on radiofrequency signals such as Wireless Fidelity (WiFi) nodes and Global System for Mobile Communications (GSM) stations [2,3]. The work in [4] evaluates other widely-used route planners (Open Street Map, Microsoft’s Bing Venue Maps), showing their indoor functionality and accuracy limitations. Other recent research solutions based on the use of the WiFi infrastructure are shown in [5,6], but the main issue of these approaches is that they require much effort in the calibration process (called fingerprinting). Furthermore, it is necessary to perform a new calibration whenever several conditions change (different furniture distribution, new WiFi access points installation, etc.). In addition, positioning error is still high (several meters), even though better than the one offered by Google Maps in indoor environments. On the other hand, Ultra-Wide Band (UWB) technologies have recently emerged as suitable solutions for indoor positioning, especially after their appearance in current smartphones, providing accuracies below decimeters. Generally speaking, UWB positioning systems are less accurate than ultrasonic ones in confined areas with LOS (Line of Sight), about 0.2 m for UWB and centimetric errors using ultrasonic (US) technology. Ultrasonic measurements degrade as the distance from the emitter to the receiver increases and, obviously, if the LOS is partially or totally blocked. Both technologies can be complementary, since UWB has greater coverage and range capacity (more than 100 m in direct LOS from elevated over-the-ground emitters). UWB emissions can partially penetrate walls and obstacles, although its accuracy and the maximum range significantly degrade in practice when operating indoors, especially due to the NLOS (Nonline of Sight) effect and the influence from metal obstacles and equipment. A complete comparison of the performances achieved by ultrasonics and UWB technologies for large indoor environments can be found in [7]. Since UWB-based systems are likely to keep improving and lowering their price due to increasing use, they will probably play a predominant role in most indoor positioning applications in coming years. Nevertheless, there is an agreement in the positioning community that different technologies may be complementary for indoor positioning. For instance, acoustic and optical solutions permit their use limited by the room extension and do not suffer interferences from outside (even malicious interference), which can provoke loss of trust and confidence in the positioning system. The coexistence of several positioning systems increases coverage, reliability and confidence.

The use of acoustic signals [1,8,9,10] to determine the position of the device allows accuracies in the range of centimeters, or even sub-centimeters in reduced areas of a few meters, with low-cost transducers. The main drawback is that an infrastructure installation is required (usually composed of several transducers) to estimate the position of the target. In recent years, several researchers have presented solutions based on ultrasonic technology for portable devices. One of the first systems is called BeepBeep [11], which measures the distance between two mobile devices by processing audible chirp signals. According to their experiments, the system provides a maximum average error of 2.7 cm for distance measurements of 12 m. The system LOK8 developed in [1] uses the smartphone’s feature of emitting inaudible sounds at a frequency near 22 kHz, thus avoiding the annoyance of audible emissions. The mobile device behaves as transmitter, whereas four microphones placed at different locations receive the signals and estimate the position of the smartphone. A similar system, called ASSIST, was developed in [12], where the smartphone generated acoustic signals from 18 kHz to 21 kHz. These are received by a set of three or more self-built receivers placed at the ceiling or walls in a room and connected to notebooks. The first low-level processing (correlation and demodulation) was carried out in the notebooks, which transmitted their data to an evaluation unit that estimated the position and sent it back to the smartphones via a cellular communication. The authors analyzed the performance of the proposed system with several commercial smartphones (IPhone, Samsung, or HTC). Nevertheless, centralized systems present some concerns about how the user location information is managed, especially in public areas [13]. In these contexts, the design of privacy-oriented systems is preferable, where the device to be located estimates its own position, instead of using a central unit. This is the case in [14], where a set of transducers emitted frequencies above the audible human limits, in the range from 19 kHz to 23 kHz, and the microphone of a smartphone was involved in their detection. A more recent proposal can be found in [15], where a linear structure of three transducers emitted low-frequency ultrasonic signals that were detected by a portable device. The reason why that range of frequencies was chosen is due to the fact that most mobile devices have a sampling frequency focused at 44.1 kHz, whereas the hardware that manages the microphone usually includes low-pass filters around 20 kHz. Nevertheless, the limitations caused by working just above the audible human range must be further analyzed to avoid potential audible artefacts. The available bandwidth in the range from 18 kHz to 22 kHz is narrow and restrains the use of spreading sequences, which are commonly employed in ultrasonic ranging systems to enable positioning in noisy multiuser environments [16,17,18,19].

In order to overcome these frequency limitations, a low-cost Ultrasonic Local Positioning System (U-LPS) was proposed in [19,20], where the beacons emitted at 41 kHz, with a 10 kHz bandwidth, and the incoming signals were not directly captured by the mobile device but rather by an attached acquisition module. Nevertheless, the coverage area of this U-LPS was limited to approximately 30 m^2^ for a height of three meters. Since people-positioning does not require decimeter accuracy in all areas, it is possible to optimize the U-LPS infrastructure deployment in order to reduce costs and calibration efforts, while increasing the localization area by merging several technologies. U-LPS can be installed only in critical zones that require decimeter accuracy; and inertial sensors can be used to obtain a coarse-grained positioning in zones where the positioning does not need to be as accurate as in narrow spaces, such as corridors. The drift errors from the inertial sensors are corrected when the mobile reaches the coverage zone of the U-LPS. This could be the case of a museum guide application, in which a coarse localization is enough when the user is walking between rooms, but decimeter positioning is desired whether he/she is approaching a specific art piece, in order to provide specific information and enhance the user experience.

For people navigation, the relative information provided by inertial sensors, such as Inertial Measurement Units (IMUs), is less accurate than the odometry information for mobile robots, but it can also be used to approximately estimate the movement of the user. Inertial sensors integrated in mobile devices are commonly used as step counters [21], as well as for other information related to physical activities. Nevertheless, the estimation of orientation is not appropriate when the position of the mobile device is not controlled or fixed at a specific position. Hence, the use of an external IMU placed on a fixed position of the person, usually the leg or over the shoe [22], offers better positioning results than handheld mobile phones, since the pedestrian’s hand often experiences movements not easy to predict [23,24,25]. For these reasons, the use of IMUs included in the smartphone was discarded in this work (although it was one of the initial considerations) due to the large errors achieved if the user did not hold the smartphone in a particular position, mainly in trajectories with several turns. Additional work can be done in the future, but some previous research on the behavior of the user regarding his/her hand movement must be also done.

In this work, a mobile application was developed to estimate the position of a user in large indoor areas by using several U-LPSs placed at key points (corridor crosses), as well as an external Bluetooth-linked inertial sensor [26] for those areas without ultrasonic coverage. The fusion of absolute information (e.g., ultrasonic signals, radiofrequency, etc.) and relative data (odometer, inertial sensors, etc.) is common for estimating the position of mobile robots [27,28,29], due to the low reliability of the odometry information. On the other hand, since the experimental were carried out in the School of Engineering of the University of Alcala [30], where indoor maps are available, a graph-matching algorithm was included that fits the obtained position to the closest point of the predefined paths by geometrical analysis. This prevents errors, such as crossing walls, in those cases with large drifts from IMUs and no U-LPS available [31,32]. The algorithms were implemented on an Android device, allowing two operation modes: a debug mode with access to IMU and ultrasonic results and normal or demo mode, where the user location is shown in real time by taking advantage of the available Google maps. Certain parts of the LOCATE-US system have been published already and will be recalled in this work, including corresponding references for further information. This is the case for the U-LPS architecture [19,33], the acquisition module [20] and a preliminary Android application [34]. Therefore, this work provides a general view of the global system, thus extending the previous work with an in-depth review of the software application, the graph-matching techniques, and a more complete experimental set-up. In summary, the main contributions are as follows.

- The proposed LOCATE-US system is described, which is intended for smartphone or any other mobile device positioning through a hybrid ultrasonic/IMU/Graph-Matching approach for wide indoor areas. IMU and graph-matching are used in zones that do not require accurate positioning, whereas the U-LPS is used to correct the drift errors from the IMU, obtaining decimeter positioning. Thus, the application can be adapted to the different granularity demands of the context.
- Any annoying audible disturbance is avoided by using ad hoc hardware to acquire the ultrasonic signals emitted at 41 kHz. Broadband signals allow simultaneous emission and robustness against noise. Time Differences of Arrival (TDOA) imply no need for additional synchronization signals.
- Real-time experimental tests are carried out, which match simulation results, obtaining a position estimate every 0.1 s. The time constraints are defined by the ultrasonic acquisition time, whereas the signal processing in the mobile device only requires 36 ms.

Compared to previous works the proposal described hereinafter presents an innovative fusion between inertial and ultrasonic sensors for positioning purposes, which allows merging the high precision provided by ultrasounds with the advantage of not having any coverage constraint from inertial sensors. If an indoor map is available, the tool includes a graph-matching algorithm to prevent errors, such as crossing walls, and provides a coarse position estimation in those cases where there is no U-LPS coverage. This is interesting in applications in which some areas require a coarse position estimation (for instance, corridors or transit zones) and others that need a more accurate position estimation (e.g., work zones). Furthermore, the proposed methods were implemented and validated on portable and commercial devices, with experimental tests, achieving accuracies and performance suitable for daily life situations.

The rest of the paper is organized as follows: Section 2 describes the available data related to the ultrasonic signals, the inertial sensor data and mapping information; Section 3 details the signal processing; Section 4 deals with the proposed implementation in Android; Section 5 explains the user interface; Section 6 shows some experimental results and, finally, conclusions are discussed in Section 7.

## 2. Available Data

**Figure 1:**
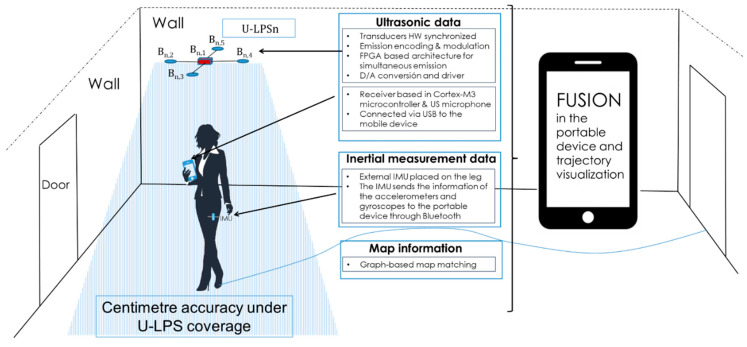
General diagram of the data to be processed in the mobile device for indoor positioning (The woman is designed by Showeet.com, accessed on 8 March 2021).

Figure 1 summarizes the data merged in the device for the sensor fusion: ultrasonic (US) data, inertial measurement data, and a model graph of the building that can be optionally included if available. This section deals with the features of the sensors used and how the raw measurements are sent to the mobile device for their processing.

### 2.1. Ultrasonic Data

**Figure 2:**
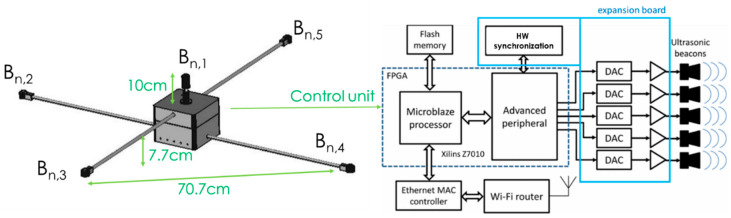
General description of the LOCATE-US beacon unit.

Obtaining the US raw data requires the deployment of the U-LPS and the acquisition hardware attached to the mobile device. Regarding the U-LPS design, it consists of five US beacons, *B*_*n*,1_ to *B*_*n*,5_, (where *n* is the index of U-LPS, since several units can be deployed to extend the coverage area) distributed in a 70.7 × 70.7 cm^2^ square structure, forming a light and easy to deploy module. It provides an approximate coverage area of 30 m^2^ at a height of three meters. Note that transducers are not coplanar, as can be observed in Figure 2, which is an advantage in the case of extending the system to 3-D positioning. Prowave 328ST160 transducers [35] were used, which have a flat frequency response between 34 kHz and 47 kHz. Thus, a bandwidth of approximately 12 kHz is available with a nearly constant phase response, which is enough for binary-phase encoding techniques [36]. Every beacon in the U-LPS is assigned with a different code with low cross-correlation among them. Since several U-LPS can be installed in the indoor space, codes assigned to beacons *B*_*n*,2_ and *B*_*n*,5_ can be repeated in different U-LPS units without common coverage area, whereas the code assigned to *B*_*n*,1_ is always unique and allows every U-LPS to be identified.

The beacons are hardware synchronized and handled by a control unit based on a System-on-Chip (SoC) architecture on a Xilinx Zynq FPGA (Field-Programmable Gate Array) device, consisting of an ARM processor and additional peripherals in charge of managing the US transmissions. The system can be configured through a wireless link from any external personal computer (PC) without uninstalling the beacon, thus avoiding a new beacon calibration every time the configuration is modified. Certain parameters, such as encoding, modulation, code length, repetition period or separation between code emissions, can be modified according to the requirements of the environment. The configured signals are stored in memory and provided to a digital-to-analog converter (DAC) at a sampling frequency of 500 kHz, which implies that a new sample is transmitted every 2 µs. The designed SoC architecture is described in detail in [33].

**Figure 3:**
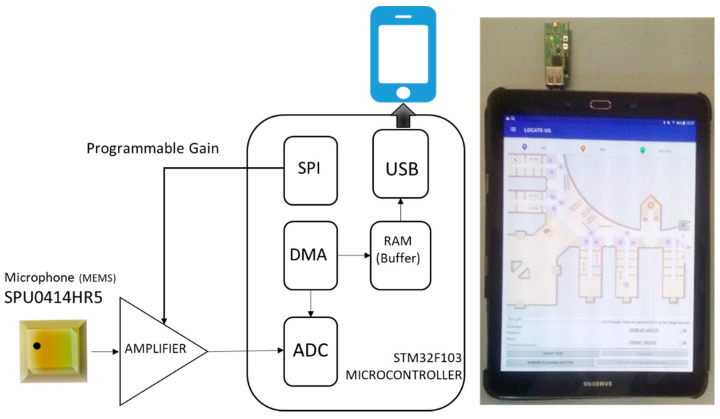
Block diagram and view of the acquisition module for LOCATE-US.

We designed an US acquisition module (48 mm × 18 mm) (see Figure 3) to acquire the signals emitted by the U-LPS. It consists of a microelectromechanical systems (MEMS) microphone (SPU0414HR5H-SB) [37] with an appropriate frequency response considering the U-LPS, which is managed by a low-cost STM32F103 microcontroller. Other components of the receiver module are a wideband amplifier, a configurable high-pass filter, and a programmable gain amplifier that allows the received signal level to be dynamically adjusted to the input range of an analog-digital converter (ADC). The acquisition module is powered through a Universal Serial Bus (USB) connection. This connection enables the sending of US raw data. The acquisition buffer is limited by the available memory in the microcontroller and can be adjusted, as can the corresponding sampling frequency. Further information concerning the US acquisition module can be found in [20].

**Figure 4:**
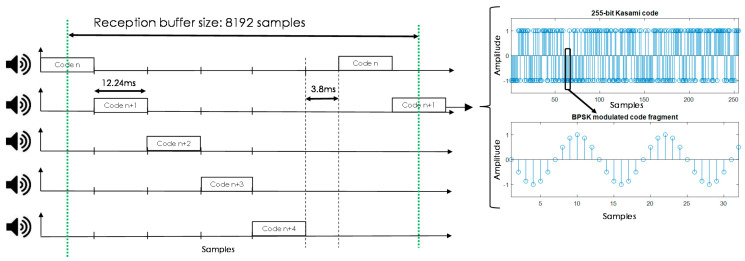
Modulation scheme and medium access technique applied to the beacon transmissions.

As mentioned, the LOCATE-US system allows the flexible configuration of US transmissions in terms of the signals to be transmitted, simultaneous or sequential emission, frequencies used and size of the acquisition buffer. All these parameters have to be set for the algorithms that run in the portable device. For clarity’s sake, considering the characteristics of the building in the tests [30], Kasami codes [38] with a length *L* = 255 bits were modulated following a Binary Phase Shift Keying (BPSK) scheme with *N_c_* = 2 periods of a sine carrier at *f_e_* = 41.67 kHz. Signals are converted at a sampling rate of *f_se_* = 500 kHz and fed into the US beacons. Kasami codes provide a suitable performance in TDOA acoustic local positioning systems, due to their low cross-correlation lobes if compared with other traditional pseudorandom codes [16,19,38]. Note that a hyperbolic TDOA positioning algorithm is considered due to the lack of synchronism between the beacons and the receiver. Besides the encoding, the results can be improved if a Time-Code Division Multiple Access (T-CDMA) is considered, where every beacon emits consecutively in a different time slot, as shown in Figure 4. Finally, it is worth mentioning that Kasami sequences and BPSK modulation have already been proven to perform suitably in a US positioning system with such bandwidth constraints, although other approaches are also feasible, such as Zadoff-Chu codes with an Orthogonal Frequency Division Multiplexing (OFDM) technique [39].

The duration of the transmission associated to every beacon was *N_c_*·*L*/*f_e_* = 12.24 ms, and the guard time between successive emission processes is set experimentally at *T_G_* = 3.8 ms, defined by the constraints coming from the hardware design. At reception, there is a downsampling factor of five, since the sampling frequency is set at *f_sr_* = 100 kHz. This factor still provides suitable performance and allows the acquisition buffer size necessary to store the incoming signals to be reduced. The buffer size is set according to the duration of the signals, the T-CDMA configuration, and considering the absence of synchronism between the beacons and the receiver. Assuming that at least six code transmissions should be acquired to properly determine the corresponding TDOAs, a minimum length in the reception buffer of 6·*M*·*L*·*f_sr_*/*f_e_* + *T_G_*·*f_sr_* = 7724 samples should guarantee at least one reception from every beacon. This length was increased up to 8192 samples to meet later specifications from the programming libraries in the final Android device.

### 2.2. Inertial Sensor Data

An IMU is an electronic device that measures and reports the accelerations and angular velocities in the three coordinate axes, since it is usually composed of a triaxial accelerometer and gyroscope. Other common components included in IMU devices are a triaxial magnetometer, to contribute to the orientation estimation, and sensors for temperature, humidity and pressure. Although all smartphones include inertial sensors, most of them are of low quality providing large drift errors [23,24,40]. In addition, if the smartphone is carried in the hand, it is difficult to get useful measurements for positioning due to the random movement of the user’s hand. In order to get accurate inertial measurements, we employed an external IMU Shimmer 3 [26] located at a fixed position in the user’s leg. The raw inertial sensor measurements (accelerometers and gyroscopes data for each axis at a sampling frequency of 100 Hz) were sent to the mobile device through a Bluetooth connection. Future work will focus on the use of the inertial sensors available in the mobile device, to compensate the user’s hand movement, so as to discard any additional hardware.

### 2.3. Map Information

A Metric Description Graph (MDG) of the floorplan from the School of Engineering in the University of Alcala previously defined in [41] was used to correct the estimated position when the inertial data are the only available information. This map is based on an Extensible Markup Language (XML) format which includes the geometric and graph description of the building in two files. The *Building.xml* file contains the coordinates of all the entities (rooms, corridors, lifts and stairs), encoded as segments with their initial and final coordinates. 

**Figure 5:**
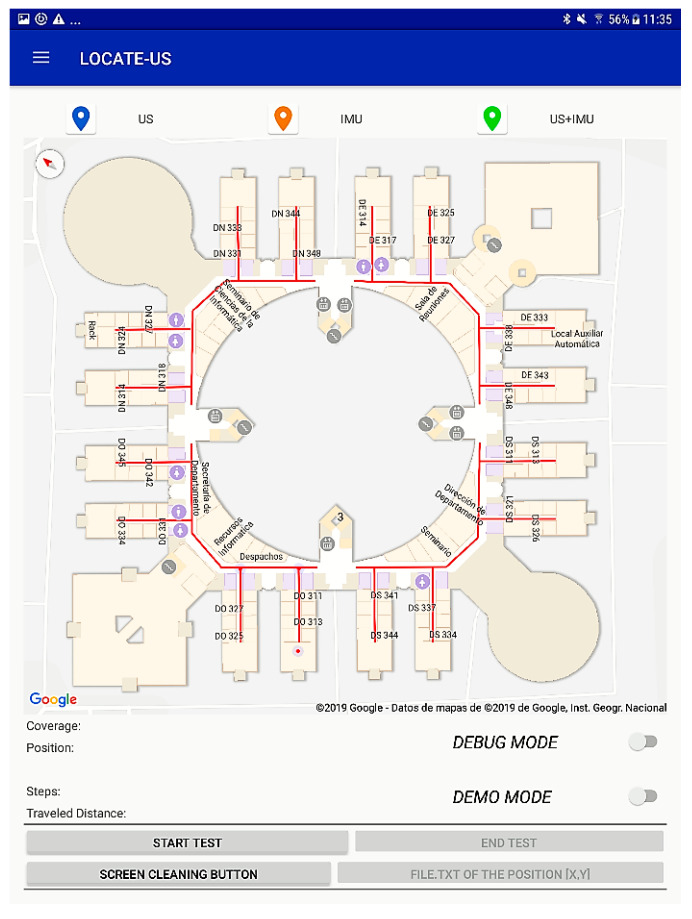
Graph considered in the LOCATE-US application for the corridors of the third floor from the School of Engineering in the University of Alcala.

The *Graphs.xml* file includes the graph information of each entity: in rooms (e.g., offices, laboratories, etc.) it is represented by the union of the room’s centroid and the middle position of the door projection on the floor, whereas the central trajectory is considered for corridors, which are encoded with the minimum set of points that compose the path. Therefore, this XML format provides a rich reference model with accurate information about the topological structure and details of different locations (room, office, laboratory, etc.). Furthermore, this XML model is integrated into Google Indoor Map Coordinates to enrich the users’ visualization experience. Google Indoor Maps will be useful in future to allow seamless transitions to outdoor spaces. Figure 5 shows a representation of the graph for the corridors on the third floor of the building displayed on Google Indoor Maps.

## 3. Processing in the Portable Device

**Figure 6:**
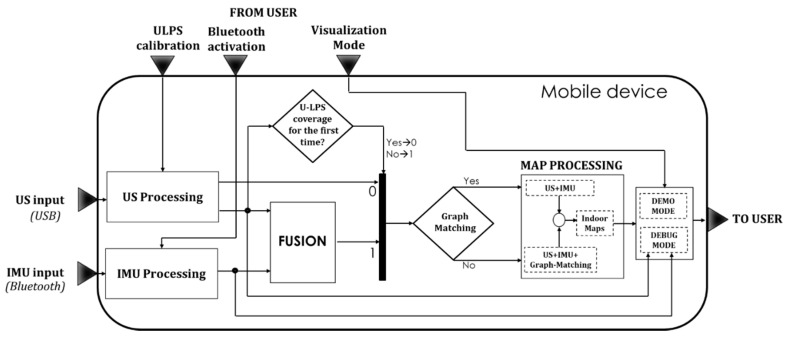
Block diagram of the LOCATE-US application.

Figure 6 summarizes the tasks implemented by the mobile device application. In general terms, US and IMU signals are processed in parallel. The first time that the ultrasonic measurements are obtained, the position is estimated directly by the US Processing block, based on a Gauss-Newton algorithm (GN). Afterwards, the FUSION block merges the US results with the IMU measurements. If the U-LPS coverage is not available, only IMU measurements are considered for the positioning. Graph-matching can be optionally included to match the estimated position to the closest one in the model graph. The obtained position is displayed on the device screen by using the Google Indoor Maps API [3]. The user can choose between normal or debug mode. The correlation results from the US signals and the inertial sensor information can be plotted in run time. Hereinafter, the main algorithms used for the US, IMU and Graph-Matching blocks are detailed, whereas their adaptation to the device app is addressed in Section 4.

### 3.1. Ultrasonic Processing

**Figure 7:**
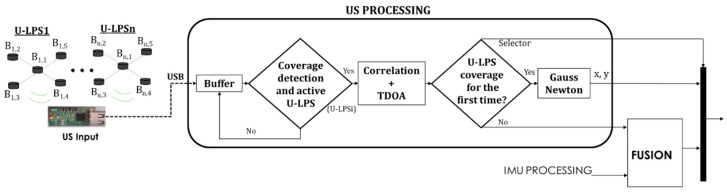
Block diagram of the US Processing tasks.

Since there is no synchronization between the U-LPS and the receiver, the US Processing block is based on the determination of TDOA between the receptions from the different beacons using the beacon received with more energy as the reference beacon. Figure 7 summarizes the US processing involved in the LOCATE-US application.

The 8192-sample buffer from the US acquisition module is read at a 10 Hz rate. A first stage (module “Coverage detection and active U-LPS” in Figure 7) searches for the U-LPS coverage by only correlating with those codes assigned to the central beacon in each unit, as indicated in (1). Note that *L* = 255 is the length of the Kasami codes, *Nc* = 2 is the number of carrier cycles, $O_{f}=\frac{f_{se}}{f_{e}}=12$ is the emission oversampling factor, *r*[*n*] is the acquired US signal and *K*_Bi,1_ is the modulated Kasami sequence assigned to the central beacon. It is worth mentioning that there is a limitation in the number *N* of possible U-LPSs to be installed, which in this case is *N* = 16 for a Kasami code with a length *L* = 255. This parameter *N* could be enough for many applications but, if required, it can be increased by choosing longer codes or other sequences with a larger family size, such as Zadoff-Chu sequences. After a certain U-LPS is identified, the correlations $C_{r,K_{B_{n,i}}}\left[k\right]$ with the codes assigned to the remaining four beacons *B_n_*_,*2*_ to *B_n_*_,*5*_ in the unit are computed, as shown in Equation (2). As a result, the number of required correlations in the US processing is reduced from 5∙*N* to *N* + 4.

(1) $$C_{r,K_{B_{n,1}}}\left[k\right]=\sum_{l=0}^{L\cdot Nc\cdot Of-1}r\left[l\right]\cdot K_{B_{n,1}}\left[l+k\right];\;1\leq n\leq N$$

(2) $$C_{r,K_{B_{n,i}}}\left[k\right]=\overset{L\cdot Nc\cdot Of-1}{\underset{l=0}{\sum }}r\left[l\right]\cdot K_{B_{n,i}}\left[l+k\right];\;2\leq i\leq 5$$

**Figure 8:**
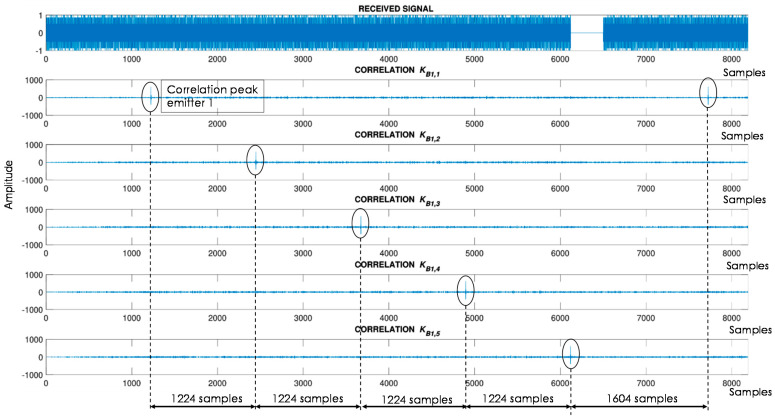
Example of correlation functions for U-LPS_1_ when the receiver’s position is equidistant to all emitters (TDOA = 0).

For the sake of clarity, an example of correlation results corresponding to the first U-LPS (U-LPS_1_) is shown in Figure 8 for a simulated situation, where the receiver’s position is equidistant to all emitters (TDOA is zero in all combinations). Considering the receiver sampling frequency *f_sr_* = 100 kHz and the T-CDMA scheme described in Section 2.1, every beacon correlation peak is separated *M*·*L*·*f_sr_*/*f_e_* = 1224 samples from the adjacent one, except for the reception from the beacons *B*_1,5_ and *B*_1,1_ that are separated 6·*M*·*L*·*f_sr_*/*f_e_* + *T_G_*·*f_sr_* = 1604 samples. The reference beacon for the TDOA estimation is the one received with the highest energy, which usually is the one closest to the receiver, thus providing a better time-of-arrival (ToA) estimation. It is worth mentioning that this approach is based on a LOS (Line-Of-Sight) situation, where the direct path is the one with most energy. If one or more of the direct paths are occluded, these measurements are not available at this moment and they are not included in the fusion filter. Intermediate situations (multipath) provoke errors that are reduced after successive measurements by the fusion filter.

**Figure 9:**
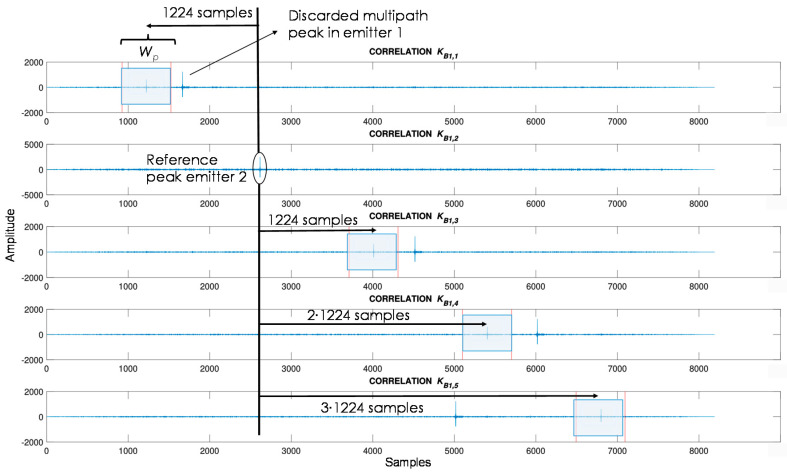
Example of correlation functions from the U-LPS_1_ with TDOA ≠ 0.

Considering the U-LPS coverage area and the beacon distribution, the distance between the reception of the closest beacon and the farthest one cannot be longer than 1 m (300 samples). Thus, those correlation peaks that occur outside the expected zone are discarded. After the higher correlation peak is detected, those corresponding to the other beacons are searched within a window of *W_p_* = 600 samples (±300 samples, before and after the ideal correlation peak, to guarantee the suitable identification of all the correlation peaks) around their corresponding ideal arrival for TDOA = 0. Figure 9 depicts an example where the receiver is placed under beacon *B*_1,2_, which is the beacon received with most energy and considered as a reference for searching the remaining beacon peaks. The analysis window *W_p_* is shadowed in blue. Note that beacon *B*_1,1_ presents a high correlation peak due to a multipath that is not selected for TDOA computation, since it appears outside the analysis window. This dynamic filter significantly reduces wrong estimations because the multipath. Also, a threshold is included to avoid the validation of noise as a correlation peak. If a beacon is not detected, the position is computed with the other four beacons, which are enough for 2-D hyperbolic trilateration. If more than one beacon is missed, the ultrasonic measurements for that acquisition are discarded.

The first time an ultrasonic signal is received, the position is estimated by means of a geometrical method, i.e., the Gauss-Newton (GN) algorithm. This estimated position is then used as the initialization position for the fusion with the inertial measurements in the next iterations of the algorithms (the user is expected to start walking from a reference point with ultrasonic coverage, in order to have an initial accurate positioning). Some examples of GN in U-LPS based systems can be found in [14,42]. If compared with other geometric methods, such as the Cayley-Menger bideterminant, GN provides a more accurate solution [43].

### 3.2. Inertial Sensor Data processing

**Figure 10:**
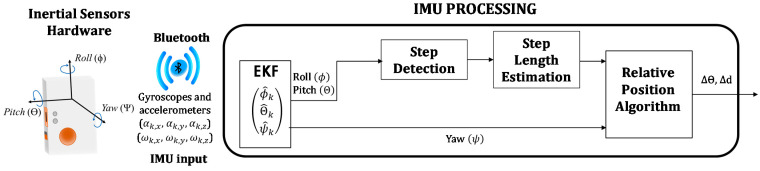
Block diagram of the inertial measurement unit (IMU) processing tasks.

Figure 10 summarizes the processing tasks in the inertial measurement unit. The portable device receives the raw inertial data from the accelerometers ($\alpha_{k,x},$ $\alpha_{k,y},\alpha_{k,z}$) in m/s^2^ and gyroscopes ($\omega_{k,x},$ $\omega_{k,y},\omega_{k,z}$) in °/s, for the three axes at every step *k*. These measurements are merged by an Extended Kalman Filter (EKF), which becomes an adequate method if measurements are contaminated with Gaussian noise [44]. This EKF provides the Euler angles of the sensor orientation, Roll (ɸ*_k_*), Pitch (ʘ*_k_*) and Yaw (*Ψ_k_*). A Step Detector algorithm checks for the maximums and minimums of the Pitch angle ʘ*_k_* to compute the number of steps. After a new step is detected, the step length $SL_{k\;}$is estimated from the absolute maximum and minimum of the pitch signal, according to Equations (3) and (4). The parameters *a_h_* and *b_h_* connect the pitch angle amplitude to the step length through a linear relationship, and they can be calculated by the universal regression proposed in [45], or experimentally. In this case, they are obtained experimentally using a reduced set of measurements and resulting in *a_h_* = 0.0294 and *b_h_* = 0.232. The position and orientation (${\hat{x}}_{k}^{-},\;{\hat{y}}_{k}^{-},\;{\hat{\theta }}_{k}^{-}$) derived from only the IMU measurements are computed according to Equation (5).

∆ʘ*_k_* = ʘmax − ʘmin (3)

*SL_k_* = *a_h_* ∗ ∆ʘ*_k_* + *b_h_* (4)

(5) $${\hat{X}}_{k}^{-}=\left[\begin{array}{c} {\hat{x}}_{k}^{-}\\ {\hat{y}}_{k}^{-}\\ {\hat{\theta }}_{k}^{-} \end{array}\right]=\left[\begin{array}{c} {\hat{x}}_{k-1}+\;SL_{k}\;\cdot \;\cos\left({\hat{\theta }}_{k-1}\;+\;\Delta \Psi_{k}\right)\\ {\hat{y}}_{k-1}+\;SL_{k}\;\cdot \;\sin\left({\hat{\theta }}_{k-1}\;+\;\Delta \Psi_{k}\right)\\ {\hat{\theta }}_{k-1}\;+\;\Delta \Psi_{k} \end{array}\right]$$

### 3.3. Measurements Fusion

The FUSION block operates when the US and IMU information are available, and if it is not the first time the receiver is inside a U-LPS coverage area. Note that for that first time the estimation of the position is obtained by the GN algorithm, as was previously described in Section 3.1. Then, the absolute orientation is determined after two successive position measurements. For the rest of the iterations the position of the receiver is estimated by using an EKF [44] that merges the ultrasonic information and the inertial measurements. To synchronize both systems, every time an ultrasonic measurement is carried out, the system captures the IMU data to feed the fusion filter. The state vector at instant k, ${\hat{X}}_{k},$ is composed by the receiver coordinates and its orientation, as stated in Equation (5).

(6) $${\hat{X}}_{k}=\left[\begin{array}{ccc} x_{k} & y_{k} & \theta_{k} \end{array}\right]$$

The prediction and update stages of the EKF are summarized as follows:

Prediction Stage:

(7) $${\hat{X}}_{k}^{-}=f\left({\hat{X}}_{k-1},SL_{k},\Delta \Psi_{k}\;\right)$$

(8) $$P_{k}^{-}=A_{k}\cdot P_{k-1}\cdot A_{k}^{T}+Q$$

Update Stage:

(9) $$K=P_{k}^{-}\cdot H_{k}^{T}\cdot {\left[H_{k}\cdot P_{k}^{-}\cdot H_{k}^{T}+R\right]}^{-1}$$

(10) $${\hat{X}}_{k}={\hat{X}}_{k}^{-}+K\cdot \left(Z_{k}-h\left({\hat{X}}_{k}^{-}\right)\right)$$

(11) $$P_{k}=\left(I-K\cdot H_{k}\right)\cdot P_{k}^{-}$$

The a priori state vector ${\hat{X}}_{k}^{-}$ is based on the previous state ${\hat{X}}_{k-1}$ and on the relative information obtained from the inertial measurements, $SL_{k}$ (step length at k) and $\Delta \Psi_{k}$ (angle increment at k). The a priori state vector is obtained as shown in Equation (5). The derivative from Equation (5) with respect to each component of the state vector corresponds to the matrix $A_{k}$, as described in Equation (12):

(12) $$A_{k}=\left[\begin{array}{ccc} 1 & 0 & -SL_{k}\cdot \sin\left({\hat{\theta }}_{k-1\;}+\Delta \Psi_{k}\right)\\ 0 & 1 & SL_{k}\cdot \cos\left({\hat{\theta }}_{k-1\;}+\Delta \Psi_{k}\right)\\ 0 & 0 & 1 \end{array}\right]$$

The matrix related to the process noise Q is given by Equation (13):

(13) $$Q=\left[\begin{array}{ccc} \sigma_{v}^{2} & 0 & 0\\ 0 & \sigma_{v}^{2} & 0\\ 0 & 0 & \sigma_{v}^{2} \end{array}\right]$$

where $\sigma_{v}^{2}$ is equal to 0.01, experimentally obtained. Then, the a priori covariance matrix $P_{k}^{-}$ is obtained according to Equation (8).

Regarding the estimation of the Kalman gain K, it is obtained by Equation (9). The observation estimations are computed by using the a priori state $vector\;h\left({\hat{X}}_{k}^{-}\right)\;$and its derivative matrix $H_{k}$, according to Equations (14) and (15), respectively.

(14) $$h({\hat{X}}_{k}^{-})=\left[\begin{array}{c} d([({\hat{x}}_{k}^{-}\;{\hat{y}}_{k}^{-}\;z_{k})],[(x_{b,2}\;y_{b,2}\;z_{b,2}])-d([({\hat{x}}_{k}^{-}\;{\hat{y}}_{k}^{-}\;z_{k})],[(x_{b,r}\;y_{b,r}\;z_{b,r}])\\ \vdots \\ d([({\hat{x}}_{k}^{-}\;{\hat{y}}_{k}^{-}\;z_{k})],[(x_{b,n}\;y_{b,n}\;z_{b,n}])-d([({\hat{x}}_{k}^{-}\;{\hat{y}}_{k}^{-}\;z_{k})],[(x_{b,r}\;y_{b,r}\;z_{b,r}])\\ \vdots \\ d([({\hat{x}}_{k}^{-}\;{\hat{y}}_{k}^{-}\;z_{k})],[(x_{b,N}\;y_{b,N}\;z_{b,N}])-d([({\hat{x}}_{k}^{-}\;{\hat{y}}_{k}^{-}\;z_{k})],[(x_{b,r}\;y_{b,r}\;z_{b,r}]) \end{array}\right]$$

(15) $$H_{K}=\left[\begin{array}{ccc} \frac{h_{1}({\hat{X}}_{K}^{-})}{\partial {\hat{x}}_{k}^{-}} & \frac{h_{1}({\hat{X}}_{K}^{-})}{\partial {\hat{y}}_{k}^{-}} & \frac{h_{1}({\hat{X}}_{K}^{-})}{\partial {\hat{\theta }}_{k}^{-}}\\ \vdots & \vdots & \vdots \\ \frac{h_{i}({\hat{X}}_{K}^{-})}{\partial {\hat{x}}_{k}^{-}} & \frac{h_{i}({\hat{X}}_{K}^{-})}{\partial {\hat{y}}_{k}^{-}} & \frac{h_{i}({\hat{X}}_{K}^{-})}{\partial {\hat{\theta }}_{k}^{-}}\\ \vdots & \vdots & \vdots \\ \frac{h_{I}({\hat{X}}_{K}^{-})}{\partial {\hat{x}}_{k}^{-}} & \frac{h_{I}({\hat{X}}_{K}^{-})}{\partial {\hat{y}}_{k}^{-}} & \frac{h_{I}({\hat{X}}_{K}^{-})}{\partial {\hat{\theta }}_{k}^{-}} \end{array}\right]$$

where $d\left(\left[\begin{array}{ccc} {\hat{x}}_{k}^{-} & {\hat{y}}_{k}^{-} & z_{k} \end{array}\right],\left[\begin{array}{ccc} x_{b,n} & y_{b,n} & z_{b,n} \end{array}\right]\right)$ is the Euclidean distance between the a priori position estimation and the $n^{th}$ beacon of the U-LPS; and $\left[\begin{array}{ccc} x_{b,r} & y_{b,r} & z_{b,r} \end{array}\right]$ are the coordinates of the beacon used as reference.

The covariance matrix R related to the Gaussian observation noise is shown in Equation (16).

(16) $$R=\left[\begin{array}{ccc} \sigma_{w}^{2} & 0.5\cdot \sigma_{w}^{2} & 0.5\cdot \sigma_{w}^{2}\\ 0.5\cdot \sigma_{w}^{2} & \ddots & 0.5\cdot \sigma_{w}^{2}\\ 0.5\cdot \sigma_{w}^{2} & 0.5\cdot \sigma_{w}^{2} & \sigma_{w}^{2} \end{array}\right]$$

where $\sigma_{w}$ is the standard deviation of the noise in the US signals, whose experimental value is 5 mm. With this low value, whether the ULPS is detected, the position given by the fusion filter is quickly set close to the position given by the ULPS, thus eliminating the accumulated error of the inertial sensor.

Finally, the position estimation *P_k_* of the receiver and the covariance matrix ${\hat{X}}_{k}$ are updated by applying Equations (10) and (11), where $Z_{k}$ contains a vector of US observations (differences of distances), and I represents a third-order identity matrix.

### 3.4. Map Representation

**Figure 11:**
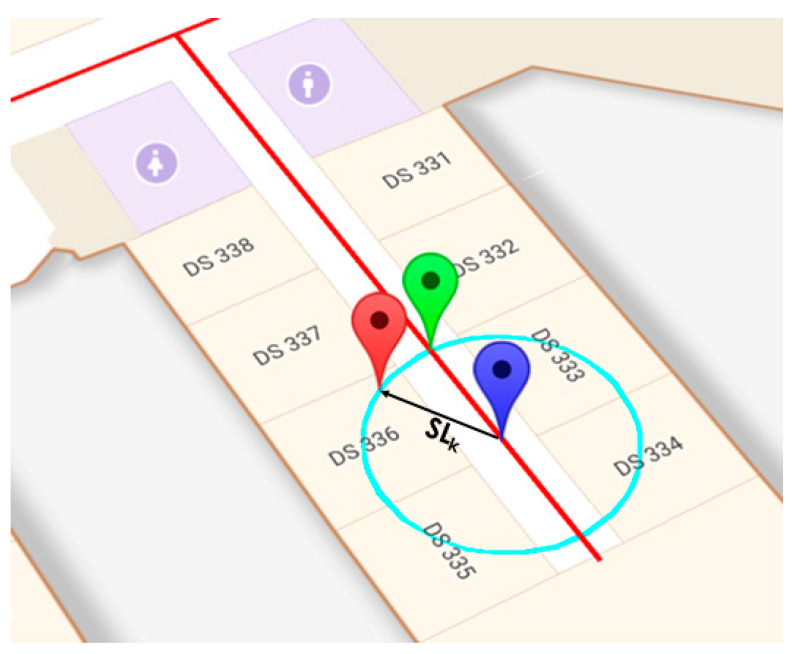
Example of graph-matching. The blue marker denotes the previous position, the red marker the new estimated one from the IMU sensors, and the green marker the one corrected by graph-matching.

Map information can also be included by means of Graph-Matching (GM) indoor localization. The aim is to prevent from situations that are not coherent, such as crossing walls, as well as to further reduce the number of U-LPS to be deployed if covering an extensive indoor area. Nevertheless, the U-LPS unit is still required in critical points, such as entrances, exits or corridor crosses, to obtain a decimeter absolute position that corrects the drift errors from the IMU, which may have led to a position in the graph far away from the actual one. The algorithm used for graph-matching consists in a geometrical estimation that matches up the estimated position from the previous stages with the position in the graph that provides the lowest error. Starting from a previously known position, the next position is obtained by drawing a circle with a radius equal to the width of the step length *SL_k_* and by calculating the intersections with the line defined in the graph. After the intersections are obtained, the new mobile device position is the one with the shortest Euclidian distance to the original estimated position (see Figure 11 for an example and [46] for more information).

Note that if graph-matching is enabled, it is only used when there is no ultrasonic coverage (to correct no feasible situations coming from the inertial sensor drift). Whether ultrasonic signals are available, the position estimation accuracy is decimetric, and it is better than the one achieved by graph-matching.

## 4. Android Implementation

**Figure 12:**
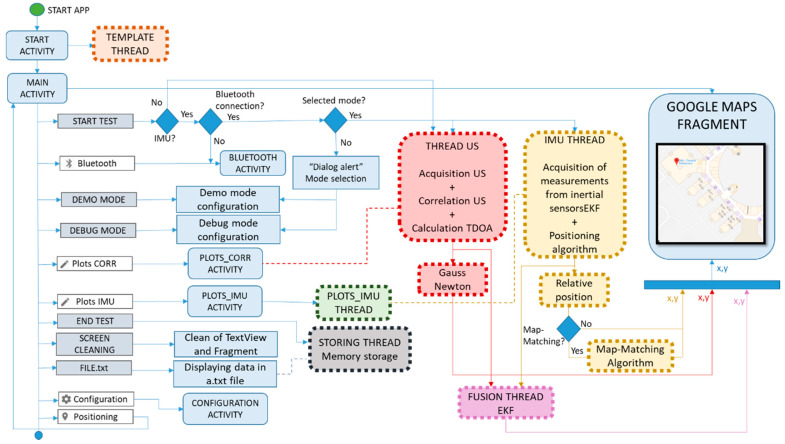
LOCATE-US Application flowchart.

Considering the selected mobile device [47], all the algorithms were implemented in an Android framework. Figure 12 shows the application flowchart, which is based on asynchronous threads. When the application is started, a *Start Activity* shows a welcome screen that runs in parallel with the *Template Thread*. This thread loads all the constants, global variables and templates to be used in the processing, such as the code patterns emitted by the beacons. The *Main Activity* controls the main App tasks, including the visualization of the user’s position on Google Indoor Maps. Other activities can be run by means of their selection in the navigation drawer. This is the case, for instance, of the *Configuration Activity* that allows inclusion of the coordinates of the U-LPS beacons deployed in the environment. If they are placed at a fixed position, they can be saved as default settings and uploaded whenever they need to be used. This allows the easy inclusion of new U-LPSs or their adaptation to possible changes. The main activity also allows the configuration of a *DEMO Mode* or a *DEBUG Mode*. In *DEBUG Mode* the activities *Plots CORR* and *Plots IMU* are available and allow the run-time visualization of the correlations with every beacon from the U-LPS involved, as well as Roll, Pitch and Yaw estimations. The *DEMO Mode* is intended for the final user and only allows the visualization of the position on the map, without further information of the aforementioned US and IMU measurements.

A *START TEST* button in the *Main Activity* launches the position estimation. The *US Thread* is in charge of processing the US signals and computes the GN algorithm. Correlations are carried out by performing an Inverse Fast Fourier Transform (IFFT) of the product between the acquired US signal and the complex conjugate of the stored modulated code patterns. On the other hand, to make use of the IMU, the user must activate this option by allowing its Bluetooth connection (*Bluetooth Activity* in Figure 12). Both threads, *IMU* and *US*, run in parallel. The *Fusion Thread* computes the EKF with the information provided by both sensors. Then, if graph-matching is allowed (in the current application the graph-matching is manually activated by the App designer), the *GM Thread* matches up the estimate with the corresponding position in the graph. Note that, since US signals provide a more accurate positioning than graph-matching, they are prioritized in the position estimation. Finally, the position is represented in real time in the device screen by using the Indoor Google Maps application programming interface in a new frame inside the main activity. All the trajectory is stored and represented in the screen since the user presses the START TEST option. In order to clean the markers from the trajectory, there is a *SCREEN CLEANING* button. Furthermore, *END TEST* allows the application to be finished and the *FILE.txt* option saves the positioning results in a TXT file.

## 5. User Interface

**Figure 13:**
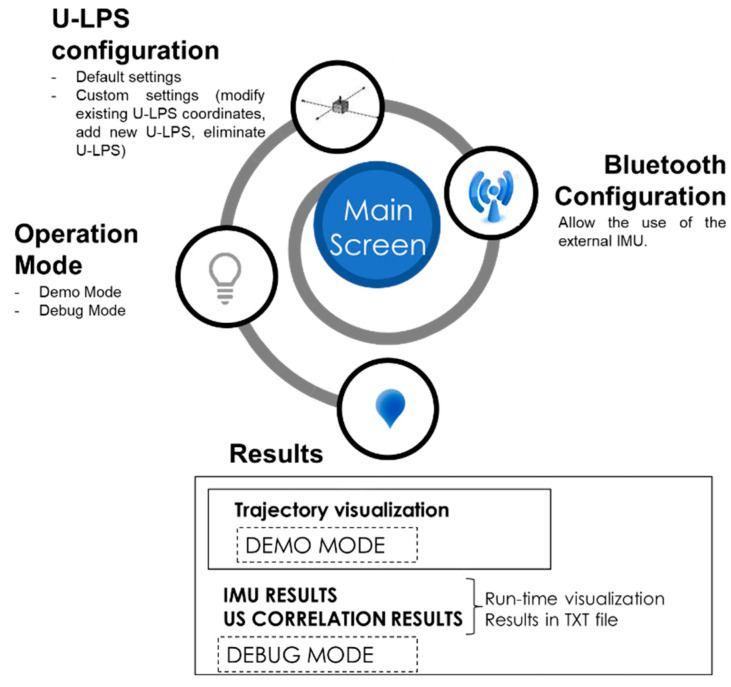
LOCATE-US user interface diagram.

A user-friendly interface was devised that easily allows different option configuration as well as displaying results and useful information (IMU, US and trajectory), while maintaining an attractive design. Figure 13 summarizes the main screens that can be found in the LOCATE-US Android Application. There is a Main Screen, where different options can be configured (IMU connection, U-LPS, operation mode). It also allows tests to be started or ended, plots the followed trajectory in the screen, and downloads results in a text file.

**Figure 14:**
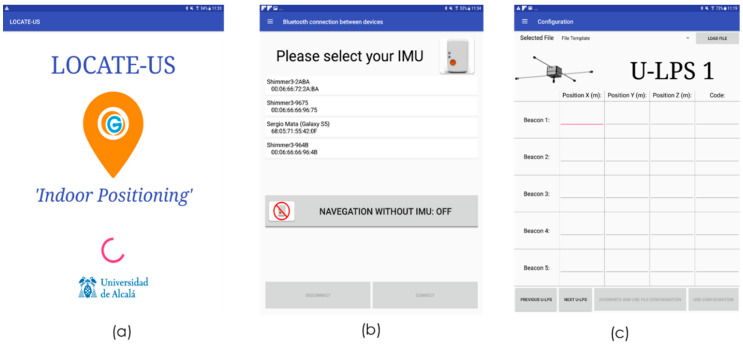
Screens from LOCATE-US application. (**a**) Welcoming screen, (**b**) Bluetooth IMU activation screen, (**c**) U-LPS configuration screen.

There is a welcoming screen (see Figure 14a), with the logo of the application and a progress bar that indicates the loading of all initial variables and templates necessary for the application. From the main menu, a navigation drawer permits the configuration of the external hardware and the visualization of IMU and US results in run time, if the *DEBUG* mode is active. Figure 14b shows the activation of Bluetooth connection to link the portable device with the external IMU. There is a list with the last IMU devices connected to facilitate their selection to the user. A Connect and Disconnect button allows use of the IMU or not. Figure 14c provides the application with the flexibility to add or modify the position of the U-LPSs deployed in the environment. For every U-LPS, the app asks for the *x*, *y* and *z* coordinates of each beacon and the code that uniquely identifies it. As mentioned before, a family with sixteen Kasami codes with a length of 255 and BPSK modulated were considered. The sixteen modulated codes are stored in internal memory and numbered from 1 to 16. Through the interface shown in Figure 14c, the code number associated to every beacon have to be included, avoiding the repetition of the code assigned to beacon 1 in any other U-LPS. Anyway, if it happens, a warning message will appear. Note that the change to any other encoding schemes involves modifying the internal templates in the Android application and the FPGA configuration in U-LPSs.

**Figure 15:**
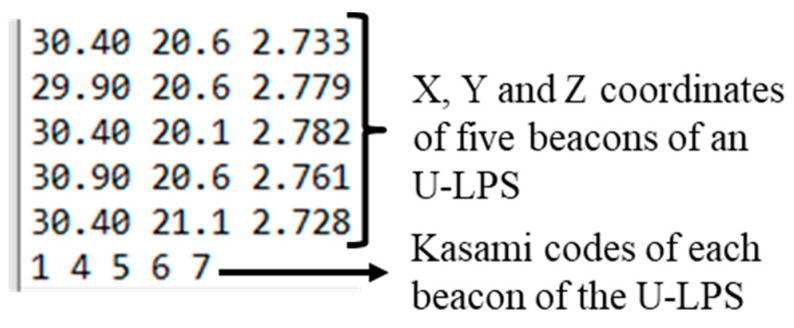
Example of plain text file for U-LPS configuration.

When the parameters for a certain U-LPS are set, the user must press “Next U-LPS” to insert them for the next U-LPS. It is also possible to make modifications to U-LPS that are already configured by going back through the “Previous U-LPS” button. After the five beacons from each one of the U-LPS deployed in the environment are configured, the user can store these specific settings by the option “Overwrite and use file configuration”. This stores the new settings as default when the application is initiated next time. If the new configuration is used, but not defined as default settings, the user must choose the option “Use Configuration”. Note that it is also possible to upload a text file with the configuration instead of using the graphical interface (see Figure 15 for an example). If no changes are required for the U-LPS configuration, this screen can be skipped, and the system operates with the default U-LPS configuration.

**Figure 16:**
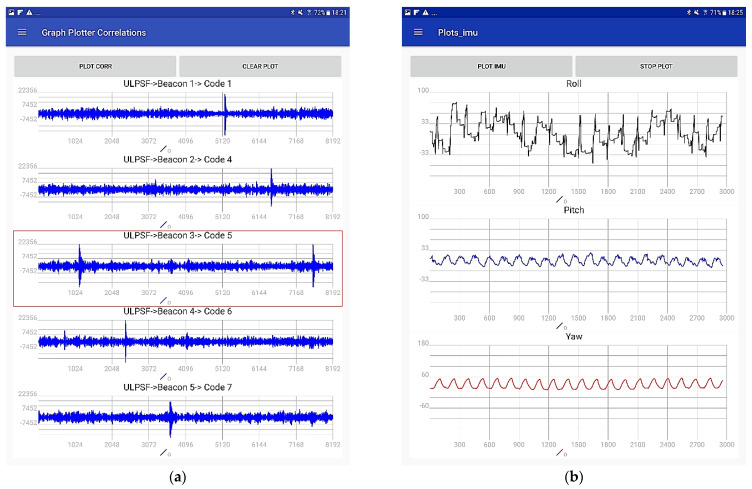
Additional screens in DEBUG mode. (**a**) US correlations, (**b**) IMU raw data.

If the *DEBUG* mode is selected, the user can see some useful data in run time. Figure 16a shows the correlation results in case of U-LPS coverage. The screen indicates the number of U-LPS from which the US signals are received (for practical issues, hereinafter, we have used letters instead of numbers to encode the different deployed U-LPSs, from A to K, instead of from 1 to 16). In Figure 16a the detected U-LPS is the *F* and the codes assigned to each beacon are 1, 4, 5, 6 and 7. It also highlights with a red box the beacon received with most energy, which is the one considered as reference in the TDOA computing. The buttons “PLOT CORR” and “CLEAR CORR” allow the correlation representation and the graphs to be cleared from the screen, respectively. On the other hand, Figure 16b allows the representation of the Euler angles from the IMU in real time, through the button “PLOT IMU”.

**Figure 17:**
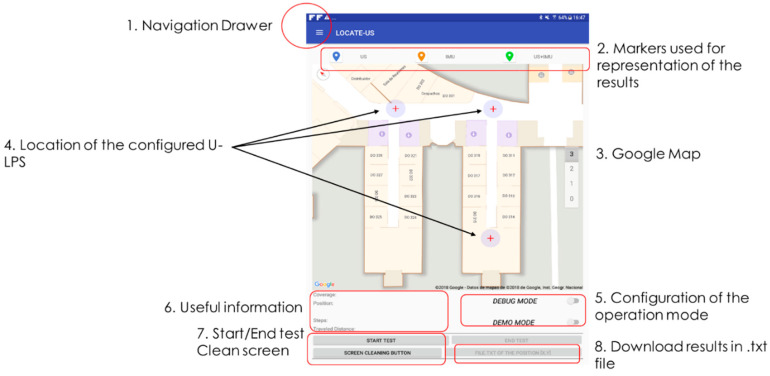
Main Screen in LOCATE-US application.

The Main Screen is shown in Figure 17. The button labelled as “1” opens a navigation drawer that allows the Bluetooth activation, U-LPS configuration and the visualization of the IMU and correlations. In “2” a legend appears with the markers used for the positioning results: in blue the coordinates obtained only with US measurements, in orange those obtained with the IMU, and in green the ones obtained after the sensor fusion and the application of the graph-matching algorithm if it is internally enabled by the designer. Note that in *DEMO* mode the results shown only correspond to those after the fusion (green mark). In “3” there is a section of the Google indoor map where the mobile device is around (it also considers the building floor). The U-LPS locations are indicated by a red cross, and their coverage area is represented by a grey circle, as shown in “4”. The operation mode is specified by two switches in “5”. Some useful information is available in “6”: if there is US coverage, it is indicated and the number of U-LPS is shown in the screen, the user’s position coordinates are also depicted, together with the number of steps since the test was initiated and the travelled distance in meters. Below (in “7”) the user can start or end the specific test, and also clean the screen (all positioning markers will be deleted). Finally, the positioning data can be downloaded into a txt file through the option indicated in “8”.

## 6. Experimental Results

**Figure 18:**
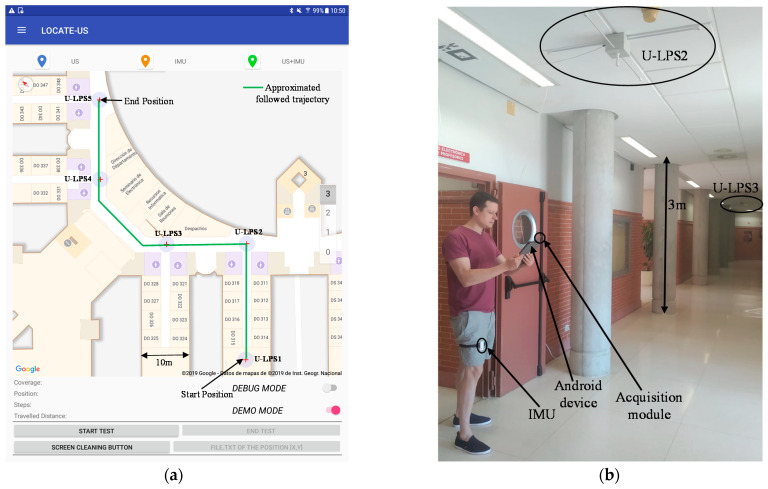
(**a**) Indoor map with the U-LPS positions and the trajectory followed by the user (in the real experiments users try to carefully follow it). (**b**) General view of the experimental setup with a person equipped with an Android device connected to the acquisition module and the IMU installed outside the pocket of the user.

The experimental tests were carried out in the School of Engineering from the University of Alcala. The positioning infrastructure was composed of five U-LPSs placed on the ceiling (around 3 m of height) at several strategic positions (one in a laboratory and the others in corridor crosses), in order to cover a distance of 57 m approximately. Figure 18a shows the indoor map with the U-LPS positions and the trajectory followed by the user. Figure 18b is an image in one spot of the trajectory where a person equipped with a tablet was located; it includes the acquisition module described in Section 2.1, and the IMU from Section 2.2 was placed outside the pocket of the user. All the installation processes were carried out and measured manually, and no calibration stage was involved. The US beacons’ positions were known (from previous tests) and the IMU was put with one axis pointing up and other pointing to the front of the person.

**Figure 19:**
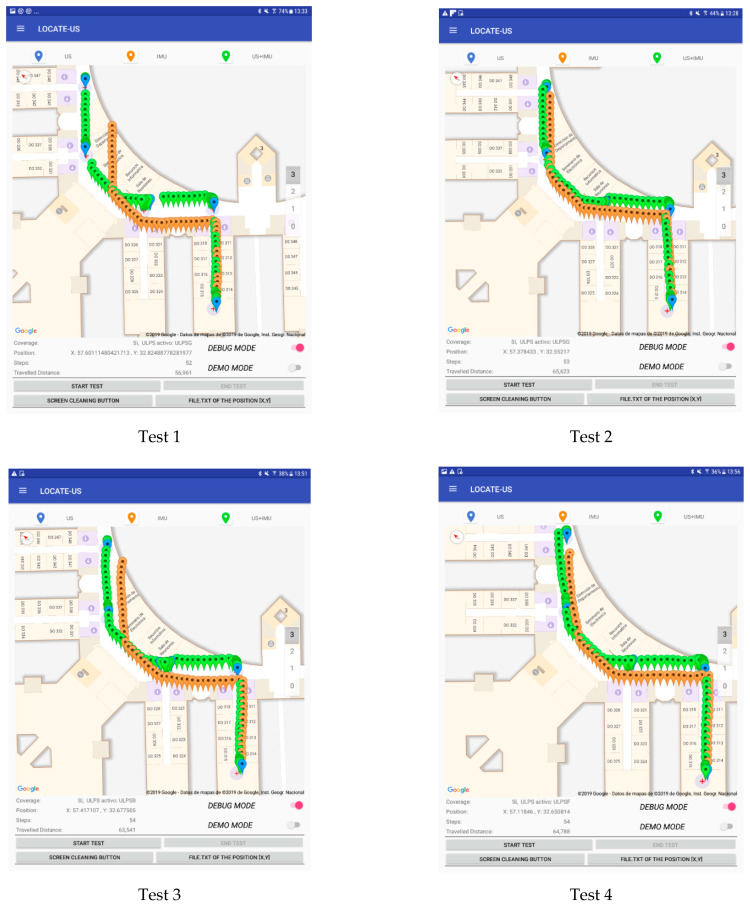
Android screen shots of the experimental tests.

The test trajectory was followed by a person with all the devices four times, following the trajectories on the floor carefully at a constant speed (normal walk), to observe the consistency of the results (no more repetitions were necessary to reach a conclusion). The map of the building could be referenced to local or universal coordinates. Figure 19 shows the visual results as screenshots of the Android device, without the use of graph-matching.

In Figure 19, the blue markers represent the first estimated position inside the coverage area of each U-LPS, the orange markers show the estimated positions by considering only the IMU measurements, and the green markers represent the fusion of the US and IMU measurements. It can be observed that fusion results are consistent, thus the errors when navigating with the IMU are suitably corrected when the user reaches a U-LPS.

**Figure 20:**
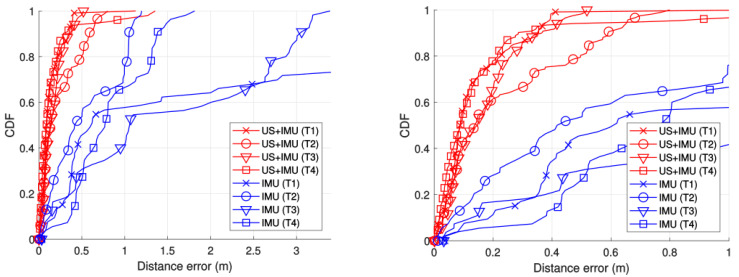
Cumulative distribution functions (CDFs) of distance errors related to the four experimental tests (**left**), comparing the IMU and the fusion (US + IMU) results, as well as a zoom for a distance error of 1m (**right**).

Figure 20 shows a Cumulative Distribution Function (CDF) of the errors in distance between each estimated position and the approximate ground truth marked around the center of the corridors. The red lines are the results of the US and IMU fusions for the four experimental tests, and the blue lines are the results when using only the information provided by the IMU.

The worst estimate related to the fusion between US and IMU achieved an error lower than 0.5 m for 80% of cases, being lower than 0.10 m for 50% of the fusion results. On the other hand, IMU estimates showed a range of errors from 1 m to 4 m in 80% of cases with a high variance in the results. Another technology in the state of the art that provides a similar decimeter accuracy is Ultra-Wide Band radio (UWB), which can be used in indoor areas. Recent news about the inclusion of an UWB module in later versions of the main brands of smartphones [48] suggests the possibility that localization services using this technology can be implemented for smartphones in near future. Taking this into consideration, we carried out a comparison between our US system and the most relevant UWB commercial system, Decawave [7], obtaining similar performance in both systems. Furthermore, there are a high number of localization systems applied to smartphones based on WiFi technology, but they provide, in general, an accuracy of some meters [49], so they are in a different accuracy range compared to the LOCATE-US system.

**Figure 21:**
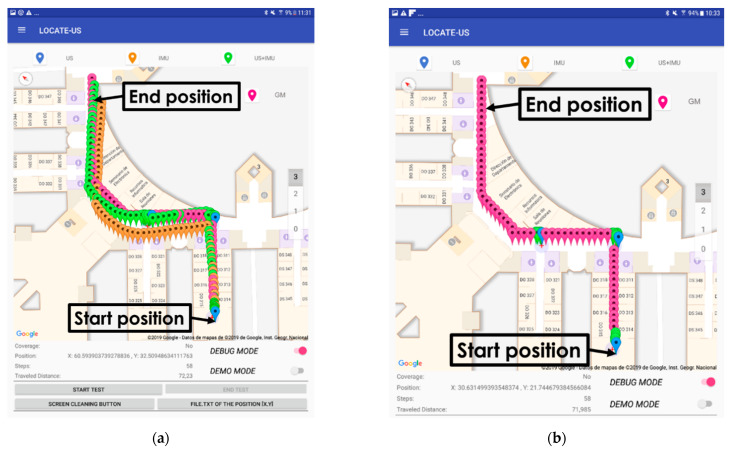
Android screenshots of the experimental test which includes the graph-matching technique. (**a**) Fusion (US+IMU), US, IMU and GM; (**b**) Fusion (US), US and GM.

If the mapping restrictions described in Section 3.4 were applied (all the estimates are represented inside a graph that considers the user walking through the middle of the corridors), the positioning errors would be zero, since the approximate ground truth trajectory is the same as the graph. In such a case, the estimates using only the IMU would be correct but, for long trajectories, the position would get lost, linked to a wrong part of the graph due to accumulative errors. Figure 21 shows the visual results of an experimental test applying the graph-matching technique when only the IMU information is available, following the same trajectory of previous experiments, but using three U-LPSs (U-LPS_1_–U-LPS_3_) instead of five, in order to observe the accumulative error in the final positions of the estimated trajectory obtained by the graph-matching method.

As can be observed, the final estimations related to the graph-matching technique are located after the real ending position. This is due to the accumulative error that the IMU information introduces in the estimation of the distance per step of the user (more details can be found in [43]). Nevertheless, this accumulative error can be corrected by using more U-LPSs in the localization area, placed at strategic positions such as entrances waypoints or corridor crosses, as for the distribution of the experimental tests shown in Figure 19. The main advantage of using the graph-matching algorithm is that all the estimates are placed in the center of each environment (e.g., center lines in corridors), filtering wrong estimates when two consecutive positions cross through walls. Another option could be the use of drift correction strategies for the IMU [50]. However, even with these corrections, the initialization of the absolute orientation is not easy, and the step model (dependent on the IMU placement) also influences in the final accumulated error, which could be too high when the trajectory has frequent turns, or the walking time is long. In such cases, some external absolute correction would always be necessary.

## 7. Conclusions

This paper presented the LOCATE-US system that combines ultrasonic signals, inertial information and graph-matching for indoor positioning with commercial mobile devices. The system allows adjustment of the accuracy granularity according to the requirements of the environment. Inertial measurements and graph-matching are used in areas that do not demand accurate positioning, whereas U-LPS are considered in zones where errors should be in the range of decimeters, in order to correct the drift of the inertial system. The designed U-LPS is modular and easy to deploy, operating at 41.67 KHz to avoid audible artefacts. An ultrasonic acquisition module with reduced dimensions was plugged into the mobile device to overcome the low-pass filters of conventional microphones in mobile devices. The manuscript describes the employed hardware, as well as the low-level processing of the ultrasonic and inertial measurements, high-level algorithms related to the information fusion, the use of maps to improve the position estimation, and the Android implementation with an intuitive user interface that plots the user’s position with Google Maps.

The system was tested in an extended indoor environment including a 57 m trajectory, where five U-LPSs were installed at strategic positions (initial and final areas of the trajectory, and corridor crosses). Experimental results showed that position estimates obtained by fusing the IMU and US information are suitable for indoor positioning, with a few decimeters of error in 80% of cases with respect to an approximate ground truth represented by the central lines in the corridors. Furthermore, the inclusion of a graph-matching method allowed estimation of the receiver position and matched it inside a valid point of the possible trajectories, similarly to a GNSS navigator, which estimates a position always inside a road. Future work will include GNSS signals to estimate the position of the user in outdoor and indoor environments with the same interface, thus providing a seamless transition between both scenarios. Additionally, LOCATE-US is prepared to evolve incorporating easily UWB readings or distance measurements from other types of sensors.

## Data Availability

Data sharing not applicable.
